# Arthroscopic versus open hindfoot fusion using a retrograde tibiotalocalcaneal nail

**DOI:** 10.1002/jeo2.70512

**Published:** 2025-11-05

**Authors:** Anna‐Kathrin Leucht, Andrea N. Veljkovic, Murray Penner, Kevin Wing, Hong Qian, Alastair Younger

**Affiliations:** ^1^ Department of Orthopaedic Surgery and Traumatology Kantonsspital Winterthur Winterthur Switzerland; ^2^ Division of Foot and Ankle, Department of Orthopedic Surgery University of British Columbia Vancouver British Columbia Canada; ^3^ Centre for Health Evaluation & Outcome Sciences University of British Columbia Vancouver British Columbia Canada

**Keywords:** hindfoot fusion, hindfoot fusion nail, tibiotalocalcaneal (TTC) fusion

## Abstract

**Purpose:**

Tibiotalocalcaneal (TTC) fusions are often performed as a salvage procedure in patients with severe hindfoot arthritis or hindfoot deformity. Comorbidities in this patient cohort are frequent, leading to increased risk of postoperative complications. Arthroscopic debridement may afford better wound healing and possible improvement of blood supply. This study compares the outcome of a challenging set of patients undergoing TTC fusion with either arthroscopic or open technique.

**Methods:**

For this cohort study, the data for patients undergoing TTC fusion from 2009 to 2018 was prospectively collected and the study design is retrospective. 58 consecutive cases were identified; in 51 cases a TTC fusion was performed while in 7 cases a tibiotalar fusion was performed in the setting of a preexisting talocalcaneal fusion. Arthroscopic technique was used in 22 fusions and open technique in 36 fusions.

**Results:**

An overall union rate of 89.7% was achieved. Five tibiotalar non‐unions and one non‐union of both the tibiotalar and talocalcaneal joints was documented. In the arthroscopic fusion group the non‐union rate was 9.1% while in the open group it was 11.1%. The overall reoperation rate was 17%. No patient in the arthroscopic fusion group required a reoperation, whereas in the open group the reoperation rate was 27.8%. In patients with PROs the AOS score improved from 53.1 to 26.2 for the arthroscopic group and from 57.2 to 32.3 for the open group. The satisfaction score improved from 1.4 to 2.7 in the arthroscopic group, and 1.1 to 2.8 in the open group.

**Conclusions:**

Arthroscopic TTC fusions are a viable alternative to the open procedure. Nonunion rates are similar, while wound complication rates and reoperation rates are lower. Outcomes measured by PROs are comparable.

**Level of Evidence:**

Level IV, case cohort study.

**Abbreviations**
AOS: Ankle Osteoarthritis Scale
ASA: American Society of Anesthesiologists
BMI: body mass index
COFAS: Canadian Orthopaedic Foot and Ankle Society
CROCS: Canadian Orthopaedic Foot and Ankle Society Reoperation Coding System
CT: computerized tomography
DM: diabetes mellitus
LFU: last follow up
MCS: mental component summary
MODEMS: Musculoskeletal Outcomes Data Evaluation and Management System
PCS: physical component summary
pre OP: preoperative
PRO: patient reported outcome
SF‐36: Short Form‐36
TTC: tibiotalocalcaneal

## INTRODUCTION

Tibiotalocalcaneal (TTC) fusion is frequently performed as a salvage procedure in patients with severe arthritis of both the ankle and subtalar joints or hindfoot deformities [11, 27, 35, 43, 45, 48]. The main surgical goal of a TTC fusion is to achieve a pain‐free, functionally stable, realigned hindfoot and ankle with a plantigrade foot [9]. Presently, the TTC fusion is most often achieved with a retrograde nail due to better biomechanical stability of the construct compared to screws, plates or external fixation, with respect to torsional and bending forces [2, 5, 6, 34, 41, 49]. Second generation of hindfoot nails have the option to incorporate compression of the fusion, which leads to higher stiffness and higher rates of union compared to earlier designs of nails [7, 36]. Hindfoot fusion with a retrograde nail is used for a wide spectrum of indications including treatment of severe deformity [23], inflammatory arthritis [3, 33, 40], fragility fractures [22, 31, 42, 51], failed prior surgery like total ankle replacement [26, 28] or ankle fusion [32], avascular necrosis [12, 17, 52] and Charcot arthropathy [17, 20, 45, 46]. The patients often have several comorbidities and are therefore prone to perioperative complications such as superficial wound infection, deep infection, and nonunion at one or both joints [8, 13, 15, 16, 24, 29, 30, 38, 44, 47].

Hindfoot fusion nails are therefore used in more demanding pathologies than isolated ankle fusions, with either bone loss, significant comorbidities leading to challenges in bone and skin healing, or a combination of both.

Arthroscopic joint preparation for fusion offers several advantages including minimal soft tissue stripping, preservation of vascularity due to less soft tissue violation. This may result in reduced wound complication [1]. Therefore the arthroscopic technique is favourable in patients with compromised vascularity, diabetes and general concerns for healing. The aim of this study was to compare the outcome of a challenging set of patients undergoing arthroscopic and open TTC fusion.

## METHODOLOGY

### Patient Enrolment

For this cohort study, we performed a retrospective chart review of patients receiving a combined ankle and subtalar fusion with a retrograde hindfoot fusion nail (VALOR^TM^ Hindfoot Fusion Nail System; Stryker) by one of 4 fellowship trained foot and ankle orthopaedic surgeons between July 2009 and August 2018 in a single center. All patients who received a hindfoot nail, and who were 21 years of age or older at the time of surgery and considered skeletal mature, were included. All patients required a TTC fusion to treat severe foot and ankle deformity including Charcot arthropathy, arthritis or instability, after failing nonoperative treatment options. Excluded from this study were patients with a conversion of total ankle replacement to fusion and patients who declined to participate.

58 patients were included in this study. Patient reported outcomes (PRO's) were available for 37 of these patients. These patients were enroled in an prospective outcomes registry for end stage ankle arthritis. This study was approved by the Institutional Review Board and the Research Ethics Board.

### Procedure selection

All surgeries were performed by 4 fellowship‐trained foot an ankle surgeons. The final decision regarding the technique of joint preparation of the ankle and subtalar joint, arthroscopic versus open, was made by the surgeon according to surgeon judgement and the patients predisposition. In addition, the use of autologous bone graft or bone graft substitute was at the surgeon's preference.

### Data collection

The baseline demographics, comorbidities and cause of ankle arthritis were recorded preoperatively. The PROs were also collected preoperatively and then annually after surgery. Union of the hindfoot fusion was assessed on plain X‐rays or CT, if performed, at the 12 weeks postoperative follow up. Reoperations were coded using the COFAS Reoperation System (CROCS), which is applicable for ankle arthrodesis and total ankle replacements [54].

### Outcome measures

The clinical outcome was assessed with the Ankle Osteoarthritis Scale (AOS) [18] and the Medical Outcomes Study Short Form‐36 (SF‐36). The AOS is a valid, self‐reported instrument that specifically measures patient symptoms and disabilities associated to ankle arthritis. The total AOS score is generated by the pain and disability subscale, each containing 9 questions. The SF‐36 [10] is a widely used health‐related quality‐of‐life measure, consisting of the physical component summary (PCS) and mental component summary (MCS) scores.

The primary outcomes of this study are the rate of revision fusion and the rate of reoperation. Secondary outcome measures were the total AOS score, with the pain and disability subscales, and the SF‐36.

### Statistical analysis

Patient demographics and clinical characteristics were summarized using descriptive statistics. Continuous variables were reported as mean and standard deviation; categorical variables were shown as frequency and percentage. All summary statistics were provided for open and arthroscopic group individually as well as for the two groups combined. Demographics and clinical characteristics were also compared between open versus arthroscopic using a two‐sample *t*‐test or Wilcoxon sum rank test for continuous variable and Chi‐square test or Fisher's exact test for categorical data when appropriate.

A linear regression model was used to compare the patient‐reported outcome scores between the two groups (arthroscopic versus open). The analyses were adjusted for age, sex, smoking status, DM, inflammatory arthritis and preoperative score.

Two‐sided *p *< 0.05 were considered to indicate statistical significance. Number and percentage of non‐union were reported. Furthermore, non‐union was analyzed with time to event analysis. Kaplan–Meier curves were used to summarize the time to non‐union by treatment group.

## RESULTS

### Demographics

A total of 58 patients received a combined ankle and subtalar fusion with a retrograde nail. The baseline demographics of all patients are illustrated in Table 1. No significant differences in baseline demographics were observed between types of hindfoot fusion (open vs. arthroscopic). The main indications for the hindfoot fusion are listed in Table 2. The retrograde hindfoot nail was used to perform a TTC fusion in 51 patients (87.9%), while 7 patients (12.1%) received a tibiotalar fusions in the setting of a previous subtalar fusion. In these 58 patients, 22 fusions were performed arthroscopically (37.9%) and 36 utilized an open technique (62.1%).

**Table 1:** Patient demographics.

Demographic	Units	Total (*n* = 58)	Arthroscopic (*n* = 22)	Open (*n* = 36)	*p*
Age at surgery	Mean (SD)	59.3 (15.0)	60.1 (12.6)	58.8 (16.5)	0.74
BMI	Mean (SD)	29 (6.8)	29.1 (6.8)	28.9 (6.9)	0.91
Gender					
F	*n* (%)	27 (46.6)	12 (54.5)	15 (41.7)	0.34
M	*n* (%)	31 (53.4)	10 (45.5)	21 (58.3)	
Diabetes	*n* (%)	16 (27.6)	7 (31.8)	9 (25.0)	0.57
Current smoking	*n* (%)	16 (27.6)	5 (22.7)	11 (30.6)	0.52

**Table 2:** Indication for surgery.^a^

Indication	Total (*n* = 58)	Arthroscopic (*n* = 22)	Open (*n* = 36)
Unspecified arthritis	32 (55.2%)	14 (63.6%)	18 (50%)
Degenerative osteoarthritis	1 (1.7%)	1 (4.6%)	0 (0.0%)
Posttraumatic osteoarthritis	6 (10.3%)	3 (13.6%)	3 (8.3%)
Inflammatory arthritis	1 (1.7%)	0 (0.0%)	1 (2.8%)
Rheumatoid arthritis	5 (8.6%)	1 (4.6%)	4 (11.1%)
Post fracture	8 (13.8%)	1 (4.5%)	6 (16.7%)
Deformity correction	25 (43.1%)	8 (36.7%)	17 (47.2%)
Osteonecrosis	3 (5.2%)	0 (0.0%)	3 (8.3%)
Infection	6 (10.3%)	0 (0.0%)	6 (16.7%)
Other	22 (37.9%)	6 (27.3%)	16 (44.4%)

a Patients may have more than one indication for surgery.

### Union/non‐union

Union of the TTC fusion was achieved in 51 patients (89.7%). One patient presented with a non‐union of both the ankle and the subtalar joint. An additional 5 patients showed a nonunion of the ankle joint (9.8%). No isolated nonunion of the subtalar joint were identified. Regarding the fusion type, a non‐union was found in 2 patients (9.1%) with an arthroscopic fusion, and in 4 patients (11.1%) with an open fusion (Table 3). There was no statistical significance between the two groups regarding union. Characteristics of the patients with nonunion are listed in Table 4.

**Table 3:** Non‐union rate by fusion type.

	Number of fusions	Non‐union	Non‐union rate
Arthroscopic	22	2	9.1%
Open	36	4	11.1%
All fusions	58	6	10.3%

**Table 4:** Characteristics of patients with nonunion.

	Age	Gender	Smoking status	BMI	Diabetes	Fusion type
1	54	F	Y	36.2	N	Open
2	54	F	N	27	N	Arthroscopic
3	47	M	Y	23.9	Y	Open
4	46	F	N	38.8	Y	Arthroscopic
5	74	F	N	24.5	N	Open
6	63	M	N	36.4	N	Open

Abbreviation: BMI, body mass index.

### Reoperation/revision

A total of 10 patients (17.2%) needed reoperations or revisions, Table 5. Some patients needed more than one surgery and more than one reoperation code was often applied. In 2 patients a reoperation was needed outside the ankle fusion. For example in one patient an additional midfoot fusion was revised. A total of 10 reoperations (17,2%) were performed due to deep infection or wound complications. All of these cases received a fusion in an open technique (27.8% of *n* = 36 open fusions). 2 revisions (3.5%) needed to be performed due to a nonunion in the absence of an infection. In these cases the approach was arthroscopically.

**Table 5:** Re‐operations summary by fusion type using the COFAS Reoperation System (CROCS).

	Re‐op Code					
	1^a^	2^b^	3^c^	7^d^	8^e^	11^f^
Arthroscopic (*N* = 22)	20 (90.9%)	0	0	0	2 (9.1%)	0
Open (*N* = 36)	28 (77.8%)	0	2 (5.6%)	10 (27.8%)	0	0
All fusions (*N* = 58)	48 (82.8%)	0	2 (3.5%)	10 (17.2%)	2 (3.5%)	0

^a^ No reoperation at or around the ankle.

^b^ Isolated hardware removal around the ankle.

^c^ Repeat operations outside the ankle fusion such as osteotomy, fusion or ligament repair and related to the fusion.

^d^ Deep infection or wound complication requiring surgical debridement.

^e^ Revision due to nonunion (no infection).

^f^ Amputation above the level of the ankle.

### Patient‐reported outcomes

**Figure 1:**
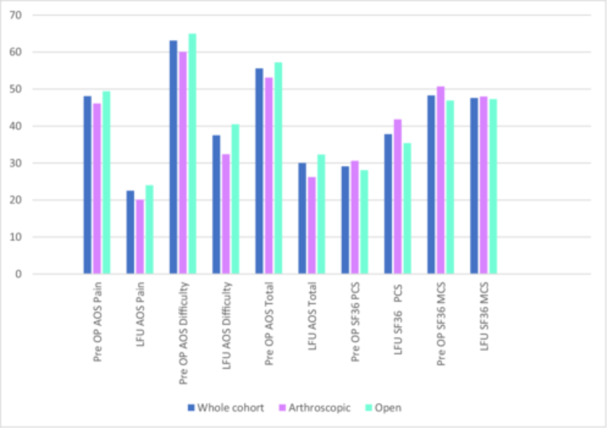
Patient reported outcome measures. PROs: AOS and SF 36, preoperative (pre OP) and at last follow up (LFU).

Figure 1 shows the AOS scores (total, pain and disability), as well as the SF‐36 PCS and MCS scores for the whole cohort and for the treatment groups (arthroscopic and open). In general, the TTC fusion led to higher outcome scores in all categories compared to preoperatively. There was no significant difference in the patient reported outcome between the arthroscopic and the open group, Table 6.

**Table 6:** Linear regression model for the outcomes scores at LFU.

Outcome	Analysis arthroscopic vs. open	Estimate mean difference	95% CI		*p* value
			Lower	Upper	
AOS Pain	Adjusted^a^	−5.71	−17.45	6.03	0.33
AOS Difficulties	Adjusted^a^	−6.6	−16.84	3.64	0.20
AOS Total	Adjusted^a^	−6.49	−16.53	3.54	0.19
SF36 PCS	Adjusted^a^	6.05	−0.07	12.18	0.05
SF36 MCS	Adjusted^a^	−0.69	−7.42	6.03	0.83

Abbreviations: CI, confidence interval; LFU, last follow up.

a Adjusted for age, sex, smoking status, DM, inflammatory arthritis and preoperative score

## DISCUSSION

TTC fusion with a hindfoot nail is a commonly performed salvage procedure in patient with severe hindfoot arthritis or hindfoot deformity. In this single‐center retrospective cohort, we were able to show that using a retrograde nail stabilization for hindfoot fusion in a high‐risk population is safe and effective, with a non‐union rate of 10.3% in 58 cases, with no significant difference between the arthroscopic and the open group. In the current literature, the prevalence of non‐union in hindfoot fusion using a retrograde nail is varying from 7% to 50% [16, 21, 25, 29, 37, 39, 47, 50, 53]. This can partially be explained by different definitions for non‐union applied in the studies. We assessed the union at the 12 weeks follow up on plain X‐rays and on CT scans, if available. We did not perform CT scans at the 3‐month follow‐up routinely, even though studies suggest that CT scans are superior in differentiating between union and non‐union [14, 19]. Similar to the study of Thomas et al. [53] if a patient did not receive revision for non‐union and the plain radiographs at 12 weeks were not suspicious of a non‐union, we considered the arthrodesis healed and the hindfoot fusion being effective.

The patient cohort in need for a hindfoot fusion often presents with several comorbidities and therefore at high risk for non‐unions. A recent meta‐analysis from Patel et al. [44] in 2021 found that a preexisting peripheral neuropathic conditions, such as diabetic neuropathy or Charcot neuropathy, is a risk factor for non‐union. Other studies have found Diabetes as a potential risk factor for non‐union [25, 29, 47]. In our cohort, 1/3 of the patients with non‐union had a co‐existing Diabetes. But we were not able to find a significant difference in survivorship in patients with diabetes or without.

In addition, we assessed the rate of re‐operation or revision surgery, which was needed in 17% of the patients in this cohort. The dominant reason for additional surgery was wound complications including infection. The systematic review of Jehan et al. [24] in 2011 analyzing a total of 641 TTC fusions found a reoperation rate of 22%.

Interestingly, all the cases in this cohort needing reoperation for infection were open fusions. However, no significant difference was found in the overall survivorship between open and arthroscopically performed hindfoot fusion. There is only sparse literature available on outcomes on arthroscopic performed hindfoot fusions with several smaller case series and one study of Baumbach et al. [4] from 2019, comparing 8 open to 15 arthroscopically performed TTC fusions. Similar to our study, reoperation for surgical site infection was only needed in the open performed hindfoot fusion group.

To the best of our knowledge, our study is the largest cohort of arthroscopic performed hindfoot arthrodesis. With a similar high union rate as open fusion, similar survivorship and reduced risk for wound infection, we were able to show that arthroscopically performed hindfoot fusion is safe and feasible. In addition, the arthroscopic group had similar outcomes to the open group with respect to PROs with similar improvement of the AOS scores and SF‐36 scores without significant difference.

## LIMITATIONS

A limitation of the present study is the retrospective study design and the lack of randomization, as well as the a heterogenous patient group. The decision whether to prepare the joints open or arthroscopically was made by the surgeon, therefore the results might be prone to bias.

The cohort size of 58 may have not had the power to appreciate a significance in reoperation rate due to infection and diabetes between open and arthroscopic techniques, although there was a trend to increased reoperation in the open group for this. In the future this could potentially be more clearly elucidated with an increased cohort size.

Furthermore, PROs were not available for the whole study cohort, hence the clinical outcome could only be assessed for a subgroup of the cohort. No exclusions were made for the patients without PROs, in favour of the larger sample size to assess surgical outcome regarding union, revision and reoperation. Another limitation to point out, is that the fusion was assessed on plain radiographs performed at 12 weeks postoperatively and not on CT, which might under‐judge the prevalence of non‐union, as discussed above.

Furthermore, there is still a necessity for a prospective study on hindfoot fusions assessing risk factors for non‐unions and inferior clinical outcomes.

## CONCLUSION

TTC fusion using a retrograde hindfoot fusion nail is a safe and effective procedure, achieving a union rate of 90% in a high‐risk patient population for non‐union with comparable results in PROs for open and arthroscopic techniques. Based on the survivorship data with revision for non‐union as end point, arthroscopic joint preparation might be advantageous to prevent postoperative wound complications. There was no significant difference regarding the survivorship between arthroscopic versus open technique or in the patients with or without diabetes.

## AUTHOR CONTRIBUTIONS

Anna‐Kathrin Leucht drafted the manuscript. Andrea N. Veljkovic helped to draft the manuscript and participated in the design of the study. Hong Qian performed the statistical analysis. Alastair Younger conceived the study and participated in its design and coordination. All authors read and approved the final manuscript. Patients were enroled and operated on by Alastair Younger, Andrea N. Veljkovic, Kevin Wing and Murray Penner.

## CONFLICT OF INTEREST STATEMENT

The authors declare no conflicts of interest.

## ETHICS STATEMENT

Ethical approval for this study was obtained from the Providence Health Care Research Institute (H19‐00605). Informed consent was performed through the ankle arthritis database.

## Data Availability

Data available on request from the authors.

## References

[1] Abuhantash M , Veljkovic A , Wing K , Gagne O , Qian H , Wong H , et al. Arthroscopic versus open ankle arthrodesis: a 5‐year follow up. J Bone Jt Surg. 2022;104:1197–1203. 10.2106/JBJS.21.01088 35793798

[2] Alfahd U , Roth SE , Stephen D , Whyne CM . Biomechanical comparison of intramedullary nail and blade plate fixation for tibiotalocalcaneal arthrodesis. J Orthop Trauma. 2005;19:703–708. 16314718 10.1097/01.bot.0000184142.90448.e3

[3] Anderson T , Linder L , Rydholm U , Montgomery F , Besjakov J , Carlsson Å . Tibio‐talocalcaneal arthrodesis as a primary procedure using a retrograde intramedullary nail: a retrospective study of 26 patients with rheumatoid arthritis. Acta Orthop. 2005;76:580–587. 16195077 10.1080/17453670510041592

[4] Baumbach SF , Massen FK , Hörterer S , Braunstein M , Waizy H , Böcker W , et al. Comparison of arthroscopic to open tibiotalocalcaneal arthrodesis in high‐risk patients. Foot Ankle Surg. 2019;25:804–811. 30455093 10.1016/j.fas.2018.10.006

[5] Bennett GL , Cameron B , Njus G , Saunders M , Kay DB . Tibiotalocalcaneal arthrodesis: a biomechanical assessment of stability. Foot Ankle Int. 2005;26:530–536. 16045843 10.1177/107110070502600706

[6] Berend ME , Glisson RR , Nunley JA . A biomechanical comparison of intramedullary nail and crossed lag screw fixation for tibiotalocalcaneal arthrodesis. Foot Ankle Int. 1997;18:639–643. 9347301 10.1177/107110079701801007

[7] Berson L , McGarvey WC , Clanton TO . Evaluation of compression in intramedullary hindfoot arthrodesis. Foot Ankle Int. 2002;23:992–995. 12449401 10.1177/107110070202301103

[8] Bibbo C , Lee S , Anderson RB , Davis WH . Limb salvage: the infected retrograde tibiotalocalcaneal intramedullary nail. Foot Ankle Int. 2003;24:420–425. 12801199 10.1177/107110070302400508

[9] Boer R , Mader K , Pennig D , Verheyen CCPM . Tibiotalocalcaneal arthrodesis using a reamed retrograde locking nail. Clin Orthop Relat Res. 2007;463:151–156. 17960678

[10] Brazier JE , Harper R , Jones NM , O'Cathain A , Thomas KJ , Usherwood T , et al. Validating the SF‐36 health survey questionnaire: new outcome measure for primary care. BMJ. 1992;305:160–164. 1285753 10.1136/bmj.305.6846.160 PMC1883187

[11] Chou LB , Mann RA , Yaszay B , Graves SC , McPeake WT , Dreeben SM , et al. Tibiotalocalcaneal arthrodesis. Foot Ankle Int. 2000;21:804–808. 11128009 10.1177/107110070002101002

[12] Cohen MM , Kazak M . Tibiocalcaneal arthrodesis with a porous tantalum spacer and locked intramedullary nail for post‐traumatic global avascular necrosis of the talus. J Foot Ankle Surg. 2015;54:1172–1177. 26002681 10.1053/j.jfas.2015.01.009

[13] Cooper PS . Complications of ankle and tibiotalocalcaneal arthrodesis. Clin Orthop Relat Res. 2001;391:33–44. 10.1097/00003086-200110000-00006 11603688

[14] Coughlin MJ , Grimes JS , Traughber PD , Jones CP . Comparison of radiographs and CT scans in the prospective evaluation of the fusion of hindfoot arthrodesis. Foot Ankle Int. 2006;27:780–787. 17054877 10.1177/107110070602701004

[15] de Cesar Netto C , Johannesmeyer D , Cone B , Araoye I , Hudson PW , Sahranavard B , et al. Neurovascular structures at risk with curved retrograde TTC Fusion nails. Foot Ankle Int. 2017;38:1139–1145. 28731802 10.1177/1071100717715909

[16] DeVries JG , Berlet GC , Hyer CF . Union rate of tibiotalocalcaneal nails with internal or external bone stimulation. Foot Ankle Int. 2012;33:969–978. 23131443 10.3113/FAI.2012.0969

[17] DeVries JG , Philbin TM , Hyer CF . Retrograde intramedullary nail arthrodesis for avascular necrosis of the talus. Foot Ankle Int. 2010;31:965–972. 21189189 10.3113/FAI.2010.0965

[18] Domsic RT , Saltzman CL . Ankle osteoarthritis scale. Foot Ankle Int. 1998;19:466–471. 9694125 10.1177/107110079801900708

[19] Dorsey ML , Liu PT , Roberts CC , Kile TA . Correlation of arthrodesis stability with degree of joint fusion on MDCT. Am J Roentgenol. 2009;192:496–499. 19155416 10.2214/AJR.08.1254

[20] Ersin M , Demirel M , Chodza M , Bilgili F , Kiliçoglu OI . Mid‐term results of hindfoot arthrodesis with a retrograde intra‐medullary nail in 24 patients with diabetic Charcot neuroarthropathy. Acta Orthop. 2020;91:336–340. 32233910 10.1080/17453674.2020.1746605 PMC8023973

[21] Ford SE , Kwon JY , Ellington JK . Tibiotalocalcaneal arthrodesis utilizing a titanium intramedullary nail with an internal pseudoelastic nitinol compression element: a retrospective case series of 33 patients. J Foot Ankle Surg. 2019;58:266–272. 30612872 10.1053/j.jfas.2018.08.046

[22] Georgiannos D , Lampridis V , Bisbinas I . Fragility fractures of the ankle in the elderly: Open reduction and internal fixation versus tibio‐talo‐calcaneal nailing: Short‐term results of a prospective randomized‐controlled study. Injury. 2017;48:519–524. 27908492 10.1016/j.injury.2016.11.017

[23] Hammett R , Hepple S , Forster B , Winson I . Tibiotalocalcaneal (hindfoot) arthrodesis by retrograde intramedullary nailing using a curved locking nail. The results of 52 procedures. Foot Ankle Int. 2005;26:810–815. 16221452 10.1177/107110070502601004

[24] Jehan S , Shakeel M , Bing AJ , Hill SO . The success of tibiotalocalcaneal arthrodesis with intramedullary nailing—a systematic review of the literature. Acta Orthop Belg. 2011;77:644–651. 22187841

[25] Jeng CL , Campbell JT , Tang EY , Cerrato RA , Myerson MS . Tibiotalocalcaneal arthrodesis with bulk femoral head allograft for salvage of large defects in the ankle. Foot Ankle Int. 2013;34:1256–1266. 23650649 10.1177/1071100713488765

[26] Johl C , Kircher J , Pohlmannn K , Jansson V . Management of failed total ankle replacement with a retrograde short femoral nail: a case report. J Orthop Trauma. 2006;20:60–65. 16424813 10.1097/01.bot.0000171880.03581.4a

[27] Kile TA , Donnelly RE , Gehrke JC , Werner ME , Johnson KA . Tibiotalocalcaneal arthrodesis with an intramedullary device. Foot Ankle Int. 1994;15:669–673. 7894640 10.1177/107110079401501208

[28] Kotnis R , Pasapula C , Anwar F , Cooke PH , Sharp RJ . The management of failed ankle replacement. J Bone Joint Surg Br. 2006;88:1039–1047. 16877603 10.1302/0301-620X.88B8.16768

[29] Kowalski C , Stauch C , Callahan R , Saloky K , Walley K , Aynardi M , et al. Prognostic risk factors for complications associated with tibiotalocalcaneal arthrodesis with a nail. Foot Ankle Surg. 2020;26:708–711. 31543311 10.1016/j.fas.2019.08.015

[30] Lee M , Choi WJ , Han SH , Jang J , Lee JW . Uncontrolled diabetes as a potential risk factor in tibiotalocalcaneal fusion using a retrograde intramedullary nail. Foot Ankle Surg. 2018;24:542–548. 29409267 10.1016/j.fas.2017.07.006

[31] Lemon M , Somayaji HS , Khaleel A , Elliott DS . Fragility fractures of the ankle: stabilisation with an expandable calcaneotalotibial nail. J Bone Joint Surg Br. 2005;87:809–813. 15911664 10.1302/0301-620X.87B6.16146

[32] Mader K , Pennig D , Verheyen CC , Gausepohl T . Minimally invasive ankle arthrodesis with a retrograde locking nail after failed fusion. Strategies Trauma Limb Reconstr. 2007;2:39–47. 18427914 10.1007/s11751-007-0018-4 PMC2321722

[33] Madezo P , de Cussac JB , Gouin F , Bainvel JV , Passuti N . [Combined tibio‐talar and subtalar arthrodesis by retrograde nail in hindfoot rheumatoid arthritis]. Revue de chirurgie orthopedique et reparatrice de l′appareil moteur. 1998;84:646–652. 9881411

[34] Means KR , Parks BG , Nguyen A , Schon LC . Intramedullary nail fixation with posterior‐to‐anterior compared to transverse distal screw placement for tibiotalocalcaneal arthrodesis: a biomechanical investigation. Foot Ankle Int. 2006;27:1137–1142. 17207444 10.1177/107110070602701221

[35] Moore TJ , Prince R , Pochatko D , Smith JW , Fleming S . Retrograde intramedullary nailing for ankle arthrodesis. Foot Ankle Int. 1995;16:433–436. 7550958 10.1177/107110079501600710

[36] Mückley T , Eichorn S , Hoffmeier K , von Oldenburg G , Speitling A , Hoffmann GO , et al. Biomechanical evaluation of primary stiffness of tibiotalocalcaneal fusion with intramedullary nails. Foot Ankle Int. 2007;28:224–231. 17296144 10.3113/FAI.2007.0224

[37] Mückley T , Klos K , Drechsel T , Beimel C , Gras F , Hofmann GO . Short‐term outcome of retrograde tibiotalocalcaneal arthrodesis with a curved intramedullary nail. Foot Ankle Int. 2011;32:47–56. 21288434 10.3113/FAI.2011.0047

[38] Mückley T , Ullm S , Petrovitch A , Klos K , Beimel C , Fröber R , et al. Comparison of two intramedullary nails for tibiotalocalcaneal fusion: anatomic and radiographic considerations. Foot Ankle Int. 2007;28:605–613. 17559769 10.3113/FAI.2007.0605

[39] Myers TG , Lowery NJ , Frykberg RG , Wukich DK . Ankle and hindfoot fusions: comparison of outcomes in patients with and without diabetes. Foot Ankle Int. 2012;33:20–28. 22381232 10.3113/FAI.2012.0020

[40] Nagashima M , Tachihara A , Matsuzaki T , Takenouchi K , Fujimori J , Yoshino S . Follow‐up study of ankle arthrodesis in severe hind foot deformity in patients with rheumatoid arthritis using an intramedullary nail with fins. Modern Rheumatol. 2005;15:269–274. 10.1007/s10165-005-0410-1 17029076

[41] O'Neill PJ , Logel KJ , Parks BG , Schon LC . Rigidity comparison of locking plate and intramedullary fixation for tibiotalocalcaneal arthrodesis. Foot Ankle Int. 2008;29:581–586. 18549754 10.3113/FAI.2008.0581

[42] Ochman S , Evers J , Raschke MJ , Vordemvenne T . Retrograde nail for tibiotalocalcaneal arthrodesis as a limb salvage procedure for open distal tibia and talus fractures with severe bone loss. J Foot Ankle Surg. 2012;51:675–679. 22621859 10.1053/j.jfas.2012.04.015

[43] Papa JA , Myerson MS . Pantalar and tibiotalocalcaneal arthrodesis for post‐traumatic osteoarthrosis of the ankle and hindfoot. J Bone Joint Surg. 1992;74:1042–1049. 1355770

[44] Patel S , Baker L , Perez J , Vulcano E , Kaplan J , Aiyer A . Risk factors for nonunion following tibiotalocalcaneal arthrodesis: a systematic review and meta‐analysis. Foot Ankle Surg. 2022;28:7–13. 33685828 10.1016/j.fas.2021.02.010

[45] Pinzur MS , Kelikian A . Charcot ankle fusion with a retrograde locked intramedullary nail. Foot Ankle Int. 1997;18:699–704. 9391814 10.1177/107110079701801104

[46] Pinzur MS , Noonan T . Ankle arthrodesis with a retrograde femoral nail for Charcot ankle arthropathy. Foot Ankle Int. 2005;26:545–549. 16045846 10.1177/107110070502600709

[47] Pitts C , Alexander B , Washington J , Barranco H , Patel R , McGwin G , et al. Factors affecting the outcomes of tibiotalocalcaneal fusion. Bone Joint J. 2020;102–B:345–351. 10.1302/0301-620X.102B3.BJJ-2019-1325.R1 32114814

[48] Russotti GM , Johnson KA , Cass JR . Tibiotalocalcaneal arthrodesis for arthritis and deformity of the hind part of the foot. J Bone Joint Surg. 1988;70:1304–1307. 3182883

[49] Santangelo JR , Glisson RR , Garras DN , Easley ME . Tibiotalocalcaneal arthrodesis: a biomechanical comparision of multiplanar external fixation with intramedullary fixation. Foot Ankle Int. 2008;29:936–941. 18778675 10.3113/FAI.2008.0936

[50] Steele JR , Kildow BJ , Cunningham DJ , Dekker TJ , DeOrio JK , Easley ME , et al. Comparison of tibiotalocalcaneal arthrodeses using a sustained dynamic compression nail versus nondynamized nails. Foot Ankle Specialist. 2020;13:193–200. 31018671 10.1177/1938640019843332

[51] Taylor BC , Hansen DC , Harrison R , Lucas DE , Degenova D . Primary retrograde tibiotalocalcaneal nailing for fragility ankle fractures. Iowa Orthop J. 2016;36:75–78. 27528840 PMC4910785

[52] Tenenbaum S , Stockton KG , Bariteau JT , Brodsky JW . Salvage of avascular necrosis of the talus by combined ankle and hindfoot arthrodesis without structural bone graft. Foot Ankle Int. 2015;36:282–287. 25377390 10.1177/1071100714558506

[53] Thomas AE , Guyver PM , Taylor JM , Czipri M , Talbot NJ , Sharpe IT . Tibiotalocalcaneal arthrodesis with a compressive retrograde nail: a retrospective study of 59 nails. Foot Ankle Surg. 2015;21:202–205. 26235861 10.1016/j.fas.2015.01.001

[54] Younger ASE , Glazebrook M , Veljkovic A , Goplen G , Daniels TR , Penner M , et al. A coding system for reoperations following total ankle replacement and ankle arthrodesis. Foot Ankle Int. 2016;37:1157–1164. 27530987 10.1177/1071100716659037

